# Double Cystic Duct: Preoperative use of MRCP Without Being Aware of the Anatomic Anomaly: A Case Report

**DOI:** 10.3389/fsurg.2022.892927

**Published:** 2022-06-27

**Authors:** Zhi Liang, Junsheng Chen, Yonggui Liang, Jijun Wang, Xiaobiao Song

**Affiliations:** ^1^ Department of General Surgery, Baotou Central Hospital, Baotou, Inner Mongolia, China; ^2^ Baotou Medical College, Baotou, Inner Mongolia, China

**Keywords:** double cystic duct, gallbladder, laparoscopic cholecystectomy, MRCP, case report

## Abstract

**Introduction:**

A biliary anomaly is occasionally encountered, however, a double cystic duct is exceedingly rare during surgery. It is pivotal for surgeons to recognize the anatomic variations in Cholangiography which is performed under fluoroscopic guidance Intraoperatively to prevent possible complications.

**Case Presentation:**

Herein, the case of a 66-year-old female patient with acute cholecystitis, in which preoperative Magnetic Resonance Cholangiopancreatograph (MRCP) did not identify a single gallbladder with double cystic ducts, is presented. Intraoperatively we identified a double cystic duct and it was safely ligated with clips. Anatomic variability was also confirmed by Cholangiography which was performed under fluoroscopic guidance. Furthermore, the patient was symptom-free through 1 year of follow-up assessment.

**Conclusions:**

In particular, when we do not identify anatomic variability based on imaging, cholangiography under fluoroscopic guidance during surgery was a powerful tool that may clearly show the anomaly of a single gallbladder with double cystic ducts.

## Introduction

Double cystic duct is a very rare anatomic variability in the extrahepatic biliary system, which can be occasionally encountered during surgery. However, there have been no studies reported to date wherein that anatomic variability arises independently in two cystic ducts with a gallbladder in the literature. In this case report, we shed light on an extremely rare case of a patient with double cystic ducts, one is a low confluence of the cystic duct (CD) to common bile duct (CBD), and the other drains into the right hepatic duct, who underwent laparoscopic cholecystectomy without being aware of the anatomic anomaly.

## Case Presentation

**Figure 1:**
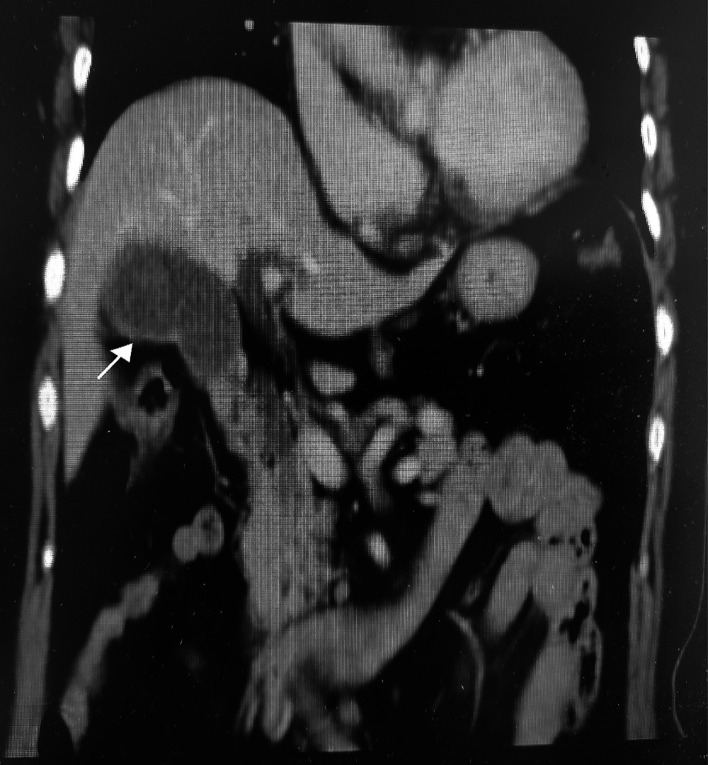
Representative coronal plane enhanced computed tomography (CT) image from a 66-year-old female patient with persistent pain for the previous 10 h, showing a typical-looking inflamed gallbladder (arrow) with marked distention and wall thickening.

**Figure 2:**
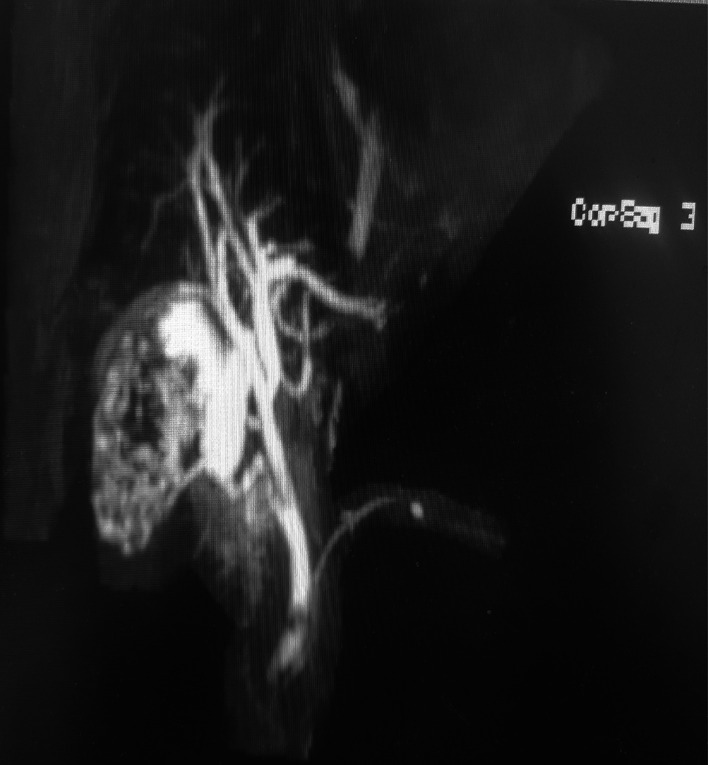
Magnetic Resonance Cholangiopancreatography (MRCP) shows the gallbladder to be filled with stones. The cystic duct is low confluence to common bile duct (CBD) in MRCP.

**Figure 3:**
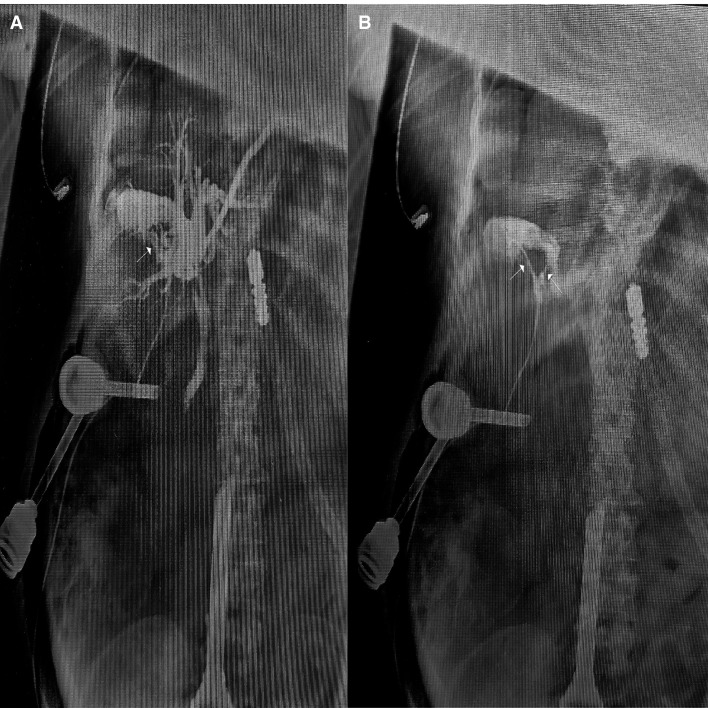
Representative cholangiogram at the supine position from a 66-year-old female patient who had presented with persistent pain for the previous 10 h, showing: the accessory cystic duct (arrow) draining into the right hepatic bile ductal system (**A**); between the inflow accessory cystic duct (right arrow) and outflow cystic duct (left arrow) of contrast agents demonstrated double cystic duct (**B**).

A 66-year-old female patient, with a history of hypothyroidism and essential hypertension, presented at the emergency room of Baotou Central Hospital in August 2021 with persistent pain for the previous 10 h. In addition, Murphy’s sign was revealed in the physical examination. The laboratory studies of the patient showed a higher white blood cell (WBC) count (16.19/L) and elevated aspartate aminotransferase (463 U/L), alanine aminotransferase (641 U/L), total bilirubin (66.5 µmol/L), direct bilirubin (40.1 µmol/L), and indirect bilirubin (26.4 µmol/L). Ultrasonography of the abdomen revealed a coarse-walled gallbladder with multiple calculi. A clinical diagnosis of acute cholecystitis was supported by computed tomography findings of gallbladder lumen distention with gall stones, moreover, there was the discovery of wall thickening in computed tomography (Figure 1). Subsequent Magnetic Resonance Cholangiopancreatography (MRCP) was performed for preoperative evaluation (Figure 2). The results revealed acute cholecystitis with multiple gall stones and calculi of the lower end of common bile duct. Additionally, the cystic duct showed a low confluence of CD to CBD in MRCP (Figure 2). Subsequently, laparoscopic cholecystectomy was performed. The liver was normal and the gallbladder was thick-walled with pericholecystic adhesions during surgery. Additionally, the cystic duct was identified as a low and wide tubular structure entering the gallbladder, which was clipped and divided. However, we found that another tubular structure was also seen arising from the neck of the gallbladder, joining the right hepatic duct. We then ligated the tubular structure with a clip, which obstructed the stones. Besides, the gallbladder was successfully isolated from the liver. Then the choledocholithiasis was removed by choledochoscope. To determine whether this was a double cystic duct connected to a double gallbladder, we performed cholangiography under fluoroscopic guidance. Foremost, identifying a double cystic duct with a clip, we open it with scissors. The results revealed the second cystic duct and a normal right hepatic duct with contrast outlining the CBD and draining into the first cystic duct. The double cystic duct with a single gallbladder hypothesis was valid (Figure 3). The operation was completed without any complications.

## Discussion

A double cystic duct with a double gallbladder is an extremely rare anomaly, accounting for fewer than 20% of published cases (1, 2). Generally, a double cystic duct has a connection with a double gallbladder (3). The earliest report containing an example of a double cystic duct appeared in 1926 (4). Thirty years later, the double cystic duct was classified into three main types by Flannery and Caster (5). (1) In the “Y” type (55%). The two cystic ducts join to form a common channel that enters the CBD. (2) In the “H” type (30%). The second cystic duct drains separately to either one of the hepatic ducts. In the “Trabecular” type (15%), an accessory duct drains directly into the liver.

In clinical practice, although the variations in cystic duct anatomy can be identified by anatomical imaging technologies including CT, MRI, and ultrasound, a double cystic duct can be difficult to recognize because it is a very rare anomaly of the extrahepatic biliary system. MRCP is reported to have a 66% sensitivity in identifying accessory bile ducts, however, it is still difficult to identify cystic duct anatomy for MRCP (6). In this case report, because the accessory bile duct was nearly completely filled with gall stones, we did not find the anatomic anomaly in the cystic duct. Therefore, in this particular setting, preoperative use of MRCP is not ideal in the present case, provided appropriate preoperative knowledge and demonstration of this biliary anomaly. Fortunately, our detection of the anatomic anomaly was timely. In addition, the results were also confirmed by Cholangiography which was performed under fluoroscopic guidance. Intraoperative identification of cholecystohepatic duct anomalies and prompt commencement of diagnoses of Cholangiography which when performed under fluoroscopic guidance is crucial to avoid surgical complications (7).

## Conclusion

In conclusion, double cystic ducts are exceptionally rare due to the absence of clinical manifestations. Hence, clinicians should be cautious in anatomic variability of the cystic duct. Simultaneously, we follow the first principles that we should not cut or electrocoagulate unclear tissue during the surgical procedure (8). This is critical to avoid anatomic variability damage as well.

## Data Availability

The original contributions presented in the study are included in the article/Supplementary Material, further inquiries can be directed to the corresponding author/s.
